# “It’s There, but Not for Us”: Pharmacy Students’ Lived Experiences of Mental Health Support at a South African University

**DOI:** 10.3390/pharmacy14060126

**Published:** 2026-08-26

**Authors:** Nqobani M. Dabengwa, Sue F. Burton, Shabnam Shaik

**Affiliations:** 1 Pharmacy Practice, Faculty of Pharmacy, Rhodes University, Makhanda 6140, South Africa; s.burton@ru.ac.za; 2 Family Medicine & Anthropology, University of the Witwatersrand, Johannesburg 2050, South Africa

**Keywords:** mental health, mental health support structures, pharmacy students, thematic analysis, phenomenology, higher education, student wellbeing, South Africa

## Abstract

This study explored pharmacy students’ lived experiences of mental health support structures at a University in South Africa. A qualitative phenomenological design was employed, with data collected through semi-structured interviews (*n* = 20) and document analysis of institutional mental health resources (2020–2024). Thematic analysis revealed five interrelated themes: awareness and visibility of support; barriers to access and utilization; institutional responses to mental health crises; cultural and contextual misalignment; and informal support and coping strategies. Although formal support services were available, students experienced them as fragmented, reactive, and insufficiently integrated into academic life. Barriers to access included long waiting times, stigma, and concerns about confidentiality. Institutional responses to crises were perceived as academically focused, with limited psychological follow-up. Students relied heavily on informal networks, including peers, family, and spirituality. These findings highlight a disconnect between institutional provision and student experience, underscoring the need for more integrated, culturally responsive, and student-centred approaches to mental health support within pharmacy education.

## 1. Introduction

Mental health challenges among university students have become a growing global concern. Universities across the world report rising levels of anxiety, depression, burnout, and psychological distress and emotional exhaustion among students, with important consequences for academic performance, personal wellbeing, professional development and retention [1,2]. While these concerns affect students across many disciplines, health professional students appear to be particularly vulnerable because of the combined demands of academically rigorous curricula, clinical placements, professional expectations, and frequent exposure to emotionally challenging situations [3,4,5].

Pharmacy students experience many of these pressures in distinctive ways. In addition to managing intensive scientific and clinical training, they must develop professional competence while navigating heavy academic workloads, frequent assessments, increasing responsibility for patient safety, and uncertainty about future employment [5,6,7]. International studies consistently report high levels of psychological distress among pharmacy students, including anxiety, depression, impostor syndrome and reduced psychological resilience with implications for academic performance, professional identity formation, and patient safety [4,8,9]. These findings have prompted growing recognition that promoting student mental health is not simply a welfare issue, but an essential component of preparing a resilient and competent healthcare workforce [10].

Much of the existing literature, however, has focused on measuring the prevalence and determinants of psychological distress using quantitative approaches. Although these studies have established the extent of mental health challenges among university students, they provide less insight into how students understand mental health, experience institutional support structures and navigate decisions about seeking help. Qualitative research offers an important complementary perspective by exploring the meanings students attribute to their experiences and the contextual factors that shape their engagement with available support services.

International qualitative studies have demonstrated that students frequently experience institutional mental health services as difficult to access, insufficiently integrated into university life and inadequately responsive to their individual circumstances [11,12]. Commonly reported barriers include stigma, concerns about confidentiality, uncertainty about available services, long waiting times, and perceptions that seeking support may threaten academic success or professional identity [4,8]. Rather than relying primarily on formal institutional services, many students turn to informal networks of peers, family members, trusted academic staff and spiritual communities for emotional support [12]. These findings suggest that the effectiveness of university mental health support cannot be evaluated solely by the existence of services, policies or programmes. Rather, meaningful evaluation requires an understanding of how these structures are experienced, interpreted and negotiated within students’ everyday academic and social lives.

Within South Africa, student mental health is shaped by additional historical, social and structural influences. The enduring legacy of apartheid, persistent socioeconomic inequality, poverty, unemployment and unequal access to healthcare continues to influence psychological wellbeing and access to mental health services [13,14,15]. Many students enter university carrying the cumulative effects of financial insecurity, family responsibilities, community violence, food insecurity and previous trauma, all of which intersect with the demands of higher education to increase vulnerability to mental distress [13,16]. These broader contextual realities influence not only the prevalence of mental health challenges but also how students understand mental health, whether they seek support and how they experience institutional responses.

The Eastern Cape presents additional challenges. Mental health services remain under-resourced and unevenly distributed, with substantial barriers to accessing appropriate care [17,18]. Although South Africa has progressive mental health legislation and universities have increasingly recognized student wellbeing as a strategic priority, significant implementation gaps remain, particularly regarding the accessibility, coordination and cultural responsiveness of institutional support systems [19]. Existing university support services are frequently described as reactive rather than preventative and often operate as isolated services rather than as components of an integrated institutional strategy [11].

Despite increasing recognition of student mental health as a priority, an important knowledge gap remains. Most South African research has focused on measuring the prevalence of mental health problems or evaluating individual support initiatives, with comparatively little qualitative research exploring how students experience institutional mental health support in their everyday lives. This gap is particularly evident within pharmacy education, where little is known about how students understand mental health, perceive available support structures or navigate help-seeking within academically demanding professional programs. Consequently, there remains a limited understanding of the relationship between institutional intentions and students’ lived experiences of mental health support.

This study addresses this gap by exploring pharmacy students’ lived experiences of mental health support at an Eastern Cape University through a qualitative phenomenological approach informed by an ethnographic lens. By integrating document analysis of institutional policies, guidelines and support resources with in-depth interviews, the study examines both the formal institutional representation of mental health support and students’ lived experiences of accessing and engaging with these structures. Combining these complementary sources of evidence provides a richer understanding of where institutional intentions align with, or diverge from, students’ everyday realities. The findings contribute to the growing body of qualitative literature on student mental health and provide evidence to inform the development of more integrated, culturally responsive and student-centred mental health support within pharmacy education and the broader South African higher education sector.

## 2. Materials and Methods

### 2.1. Study Design

This study adopted a qualitative phenomenological research design situated within an interpretivist paradigm. Phenomenology was selected to explore the meaning that pharmacy students attribute to their experiences of mental health support within the university environment and to understand how these experiences are perceived, interpreted, and embodied in daily student life [20,21]. Although phenomenology provided the primary methodological orientation, an ethnographic lens informed the broader interpretation of the findings. This enabled individual experiences to be understood within the wider institutional, cultural and policy context in which they occurred. Rather than examining participants’ experiences in isolation, the study explored how institutional practices, organisational culture and formal support structures shaped students’ perceptions and engagement with mental health services.

**Figure 1:**
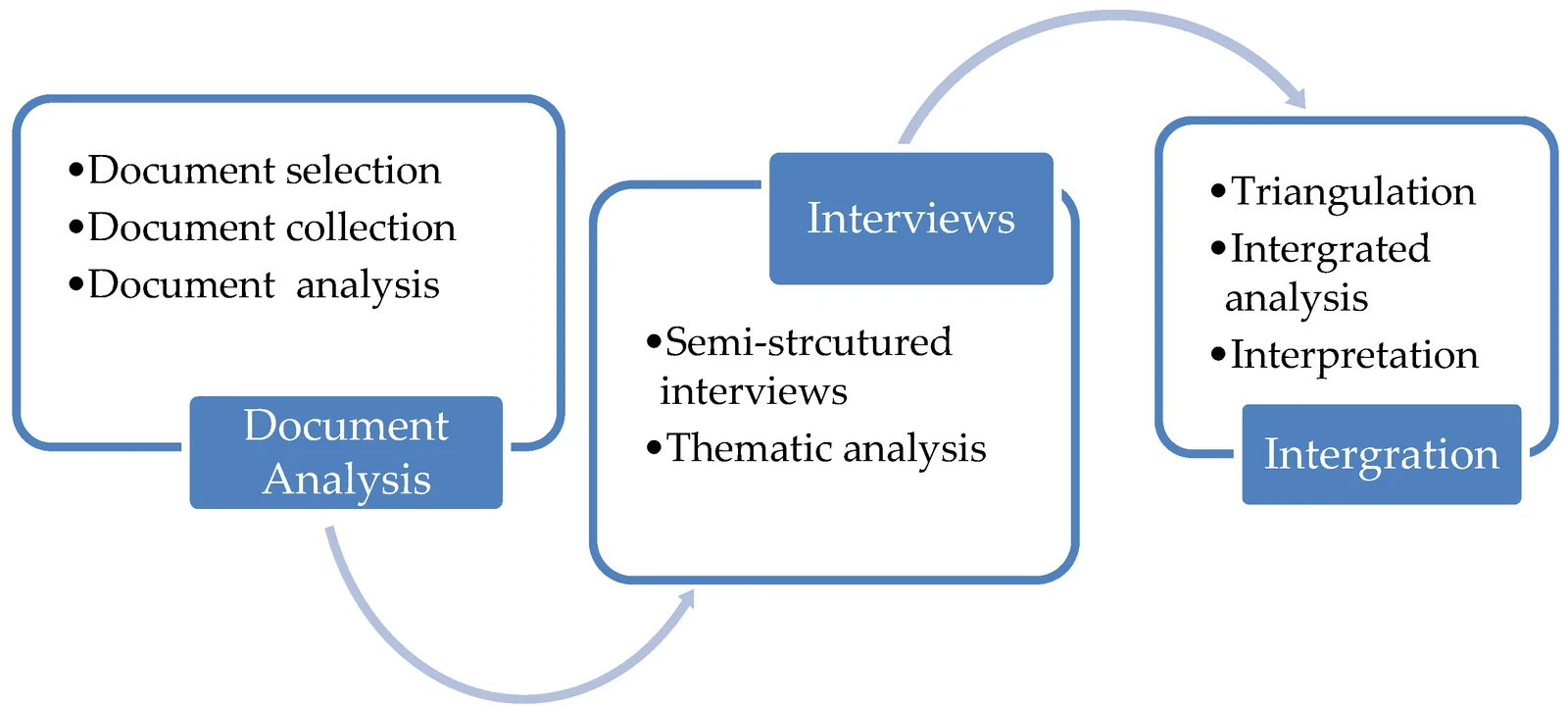
A schematic of the research design process.

To provide a richer understanding of these experiences, two complementary sources of data were collected: (i) institutional documents relating to mental health support and (ii) semi-structured interviews with pharmacy students. Integrating these data sources enabled methodological triangulation by comparing institutional representations of mental health support with students’ lived experiences, thereby strengthening the credibility and depth of interpretation (Figure 1).

### 2.2. Study Setting

The study was conducted at a public university located in the Eastern Cape Province of South Africa. The university is a historically white institution that has undergone substantial transformation since South Africa’s democratic transition and now serves a culturally and socioeconomically diverse student population.

The Faculty of Pharmacy offers undergraduate and postgraduate programs that are academically demanding and include both theoretical and experiential learning. At the time of the study, formal mental health support was primarily provided through the University Counselling Centre, Student Wellness Services and the Student Mental, Academic and Social Health (SMASH) programme. Although these services were available, the university did not have a standalone institutional mental health policy, making it an appropriate setting in which to explore how students experienced existing support structures.

### 2.3. Participants and Sampling

A purposive sampling strategy was used to recruit pharmacy students who could provide rich and varied accounts of their experiences of mental health support [22]. Maximum variation sampling was employed to ensure diversity across year of study, gender, nationality and residential status.

Eligible participants were registered undergraduate or postgraduate pharmacy students aged 18 years or older who were willing to discuss their experiences and perceptions of mental health support within the university environment.

Participants were recruited through faculty communication channels, electronic notices and student networks. Snowball sampling was not used. Twenty students participated in the study, comprising undergraduate students from all four years of the Bachelor of Pharmacy programme, as well as postgraduate students.

Data collection and analysis occurred concurrently, allowing emerging concepts to inform subsequent interviews. Data saturation was monitored throughout the analysis process. Following the eighteenth interview, no new codes or concepts were identified, and two additional interviews were conducted to confirm the sufficiency of the themes. As these interviews generated no substantially new information, data collection was concluded, indicating that adequate thematic saturation had been achieved [22,23,24].

### 2.4. Researcher Characteristics and Reflexivity

The interviews were conducted by the principal researcher, a Master of Pharmacy student with a professional background in pharmacy. While the researcher shared the broader educational context of the participants, he did not occupy a formal teaching or assessment role that could influence participation or responses.

Recognising that qualitative research is inherently interpretive, reflexivity was incorporated throughout the study. The researcher maintained reflexive memos during data collection and analysis to document assumptions, emerging insights and potential biases. Regular discussions with the supervisory team, comprising researchers with expertise in pharmacy practice and anthropology, encouraged critical reflection on coding decisions, theme development and interpretation. These discussions promoted analytical rigour and reduced the likelihood that findings reflected the perspectives of a single researcher.

### 2.5. Data Collection

Data was collected between September 2024 and February 2025 using two complementary methods.

**Phase 1: Document analysis**

Nine institutional documents published between 2020 and 2024 were purposively selected because they described or informed student mental health support at the university. Documents included university policies, strategic plans, student wellness resources, counselling service information, crisis response guidelines and other publicly available institutional documents relevant to student wellbeing.

Document analysis provided a contextual understanding of how mental health support was formally represented within the institution and informed the interpretation of students’ experiences during the interview phase.

**Phase 2: Semi-structured interviews**

Semi-structured interviews were conducted using an interview guide developed from the literature review, the study objectives, and the theoretical framework.

Before commencing the main study, the interview guide was piloted with three pharmacy students who met the inclusion criteria but were not included in the final sample. Minor refinements were made to improve question clarity, sequencing and interview flow. No substantive changes to the content occurred following the pilot interviews.

The interview guide explored students’ understanding of mental health, awareness of institutional support services, experiences of accessing support, barriers and facilitators to help-seeking, perceptions of institutional responsiveness, cultural influences and personal coping strategies. The complete interview guide is provided as Supplementary Materials.

Interviews lasted between 20 and 60 min and were conducted in private locations selected to ensure confidentiality and participant comfort. With participants’ written consent, all interviews were audio-recorded and transcribed verbatim before analysis.

### 2.6. Data Analysis

Document and interview data were analysed using six-step thematic analysis as described by Braun and Clarke, and it was supported by ATLAS.ti^®^ software (version 25.1.1) [25].

Document analysis was undertaken first using an inductive approach to identify how mental health support was represented within institutional documentation. Documents were read repeatedly, coded systematically and organized into preliminary categories describing institutional priorities, available services and organisational approaches to student wellbeing.

Interview analysis combined both deductive and inductive coding. Initial deductive codes were informed by the study objectives, interview guide and concepts emerging from the document analysis. During repeated readings of the transcripts, additional inductive codes were generated to capture unexpected ideas and experiences not reflected in the preliminary coding framework.

Coding was therefore iterative rather than linear. As analysis progressed, codes were continuously refined, merged or expanded through constant comparison across participants and between interview and documentary data. These iterative comparisons enabled the identification of broader patterns and relationships, culminating in the development of overarching themes that reflected both institutional representations and students’ lived experiences.

Methodological triangulation occurred during interpretation rather than solely during coding. Themes generated from the interviews were systematically compared with findings from the document analysis to identify areas of convergence, complementarity and divergence. This process enabled the study to distinguish between institutional intentions and students’ experiences of mental health support, thereby strengthening the credibility and interpretive depth of the findings.

### 2.7. Trustworthiness

The trustworthiness of the study was enhanced using Lincoln and Guba’s criteria of credibility, dependability, confirmability and transferability [26].

Credibility was strengthened through methodological triangulation, pilot testing of the interview guide, prolonged engagement with the data and participant verification of preliminary findings. Dependability was supported through detailed documentation of methodological decisions and maintenance of an audit trail. Confirmability was enhanced through reflexive memo writing and regular supervisory discussions regarding coding and interpretation. Transferability was facilitated by providing rich descriptions of the study context, participants and institutional setting, enabling readers to determine the applicability of the findings to similar educational environments.

### 2.8. Ethical Considerations

Ethical approval was obtained from the University’s Human Research Ethics Committee (Approval No. 2024-8031-9040). Written informed consent was obtained from all participants. Anonymity and confidentiality were strictly maintained through the use of pseudonyms and coded transcripts. Participants were informed of their right to withdraw at any point without consequence.

## 3. Results

* The direct quotes in this section come directly from student interviews, and to keep their identities anonymous, they were given codes whose key is available below Table 1.

Twenty pharmacy students participated in the interview component of the study as described in Table 1:

Findings from the interviews were interpreted alongside an analysis of nine institutional documents relating to student mental health support. Together, these complementary data sources provided insight into both the institutional framing of mental health support and students’ lived experiences of engaging with these structures. Five interrelated themes emerged from the integrated analysis.

Although the five themes are presented separately for clarity, considerable overlap existed between them:

- Awareness and visibility of support
- Barriers to access and utilization
- Institutional responses to mental health crises
- Cultural and contextual misalignment
- Informal support and coping strategies

### 3.1. Awareness and Visibility of Mental Health Support

Analysis of the institutional documents indicated that the University had established several formal mental health support structures, including the University Counselling Centre, Student Wellness Services, the Student Mental, Academic and Social Health (SMASH) programme, and crisis response pathways. Collectively, these documents portrayed mental health support as accessible and available through multiple entry points, suggesting that students could readily identify and access appropriate services when needed.

However, students’ lived experiences presented a markedly different picture. Although most participants were aware of the University Counselling Centre, they described mental health support as largely invisible in everyday university life. Support services were perceived as existing “in the background” and becoming visible only during periods of crisis, rather than being integrated into the routine academic experience. Many participants felt that mental health was regarded as a personal responsibility rather than a shared institutional priority.

One participant described how academic achievement appeared to take precedence over students’ psychological wellbeing:

“I feel like for lecturers, their primary goal or the primary work that they focus on is for us to know the work. I’m not going to lie, they would want to be assured that we know the work, but mentally, they wouldn’t really, you know, make sure that we are okay. I feel like, for them it’s just up to you that you must be okay mentally … for them it’s just like you must understand the work”

UG1-2

Several participants also felt that the level of pastoral and academic support diminished as they progressed through the programme, despite the increasing academic and emotional demands they experienced. One student reflected:

“Just because we’re pharmacy students doesn’t mean that the extra support that we have in the first year has to stop in the other years, you know, because I feel like that’s also something that just makes people tip into everything because once you see your marks decline, you don’t have everything going well, then you just slowly but surely without even realising it tap into just the dark space and everything”

UG3-3

These accounts suggest that, despite students’ awareness of formal services, mental health support was experienced as peripheral rather than as an integral component of university life. This finding contrasts with the institutional narrative presented in the document analysis. While university documents consistently emphasized the availability of counselling, wellness programs and referral pathways, there was limited evidence of strategies aimed at embedding mental health promotion within routine teaching, learning or student engagement. Mental health support, therefore, appeared to be positioned primarily as a service to be accessed when problems arose, rather than as an ongoing aspect of student wellbeing.

This disconnect was further reinforced by the absence of a clearly articulated institutional mental health policy. Rather than presenting a coordinated framework for student mental health, the document analysis suggested that support services were distributed across multiple departments and programs, each operating independently. Consequently, although a range of support mechanisms existed, they lacked the visibility and coherence necessary to foster students’ perceptions of an integrated and accessible mental health support system.

### 3.2. Barriers to Access and Utilization

Institutional documents described counselling and mental health services as accessible through clearly defined referral pathways and outlined procedures for students seeking support. However, while these documents detailed how services could be accessed, they provided little information regarding practical barriers to utilization, such as service capacity, waiting times, or the social and psychological factors that influence help-seeking. There was also limited acknowledgement of concerns relating to stigma, confidentiality or students’ perceptions of mental health services.

In contrast, participants described numerous barriers that limited their willingness or ability to engage with formal support structures. Although students recognized that services were available, they frequently perceived them as difficult to access because of long waiting times, limited appointment availability and uncertainty regarding confidentiality. One participant described the frustration of experiencing prolonged delays in accessing counselling services:

“In first year I booked an appointment to see the psychologist and it took very long, like for them to respond even like to gather confirmation of my appointments like imagine like you’ve been like wanting to have like the appointment from first term only for you to get like the actual session towards the end of the year.”

UG4-3

For some students, perceptions of poor accessibility discouraged help-seeking before they had even attempted to access services. As one participant explained:

“I do stay on social media and people always complain about having or finding it hard to access these kinds of services, so I don’t think I even try after seeing that I’m like, you know, it’s disheartening”

UG3-5

These practical barriers were further compounded by the stigma surrounding mental health, which emerged as one of the most significant deterrents to seeking professional support. Participants described deeply embedded community beliefs that associated mental illness with being “crazy” or “going mad,” creating fear and reluctance to access counselling services.

One participant reflected:

“People think having the symptoms means you are ‘crazy’, they use words like ‘crazy’ and all of that”

UG3-6

Similarly, another explained how these attitudes had shaped their own perceptions of counselling:

“… from how I was raised, you’d hear when they’re talking about someone who had to go for therapy like he was going mad, so like they had to go there.”

PG2

Together, these findings suggest that access to mental health support is shaped by more than the physical availability of services. While institutional documents presented access as a relatively straightforward process, students’ experiences demonstrated that practical, cultural and psychological barriers substantially influenced whether support was sought or utilized. The limited attention given to these experiential barriers within institutional documentation suggests that existing support frameworks may overemphasize structural availability while under-recognizing the complex realities that shape students’ help-seeking behavior. Consequently, many participants described delaying or avoiding accessing formal mental health services, even when experiencing significant psychological distress.

### 3.3. Institutional Responses to Mental Health Crises

Institutional documents outlined clear procedures for responding to students experiencing mental health crises. These included referral pathways to counselling and wellness services, mechanisms for academic accommodation, and processes intended to ensure student safety and academic continuity. The documents reflected an organized institutional response to acute mental health concerns and aligned with the university’s responsibilities for student welfare and risk management.

Students, however, described these responses as predominantly reactive and procedurally driven, with support often centred on managing the immediate academic consequences of psychological distress rather than addressing students’ longer-term mental health needs. Academic concessions, such as deadline extensions or assessment adjustments, were viewed as helpful in the short term but were not perceived as substitutes for sustained psychological care.

One participant reflected on the uncertainty surrounding help-seeking and the type of support ultimately received:

“Yeah, like sure we’re told guys if you’re struggling you can just tell us about it or voice it out … but then the thing is, who do we speak out to? Which channel are we voicing ourselves to? And is it safe enough for me to even open up? And even if I do open up, will I get the help that I need? Or will it just be a thing of, okay, we understand that you’re going through this, so we’ll just try and make leeway for you throughout the degree, which doesn’t really help.”

UG3-3

Participants also described limited continuity of care following an initial crisis. Several students felt that once the immediate situation had been addressed, there was little ongoing psychological support or structured follow-up to assist their recovery or reintegration into academic life.

As one participant explained:

“That was draining because you’re working with different people. The psychologist that you saw last year is not the one who’s here; this one doesn’t know your history, and now you have to keep on sending emails”

UG3-6

Taken together, the document analysis and interview findings reveal an important distinction between institutional preparedness and students’ experiences of care. While institutional documentation demonstrated clearly defined crisis response procedures and mechanisms to support academic progression, there was limited evidence of structured follow-up, continuity of care or reintegration support after the immediate crisis had passed. Students similarly perceived the institutional response as focused on resolving acute situations and maintaining academic continuity, rather than providing sustained psychosocial support. This convergence between the two data sources suggests that existing support structures may prioritize crisis management over longer-term recovery, leaving some students feeling unsupported once the immediate crisis has subsided.

### 3.4. Cultural and Contextual Misalignment

Institutional documents consistently emphasized principles of inclusivity, equity and student wellbeing, presenting mental health support as universally available to all students. However, these documents made limited explicit reference to how mental health services were adapted to accommodate students’ diverse cultural, linguistic, spiritual and socio-economic backgrounds. The language used throughout the policies and support materials reflected largely standardized approaches to mental health care, with little evidence of strategies to promote culturally responsive or contextually grounded service delivery.

Students’ lived experiences revealed a different perspective. Many participants described feeling that formal mental health services did not adequately recognize or engage with the realities that shaped their experiences of mental health. Cultural identity, language, socioeconomic circumstances and spirituality were viewed as important influences on wellbeing, yet participants felt these dimensions were often overlooked within formal support encounters. As a result, some students experienced a sense of disconnection from institutional services and questioned whether counsellors fully understood their lived realities.

One participant described the difficulty of establishing a therapeutic relationship when there was little shared understanding of their background:

“…I feel like my main issue with my university-appointed therapist was that there was no familiarity whatsoever. We had no common ground to the point where she thought of like recommending it to someone else”

PG3

In contrast, participants spoke positively about encounters where they experienced cultural or linguistic connection with a mental health professional. These interactions fostered greater trust, openness and a stronger sense of being understood.

As one participant explained:

“… it was much better cause she was a black woman so I didn’t have to feel like now I have to filter certain things because I can easily speak to her in my own language. I even told her that it was very helpful because I wasn’t restricted with my language.”

UG3-4

The integration of documentary and interview data highlights an important disconnect between institutional aspirations and students’ lived experiences. Although institutional documentation promoted inclusive student support, there was limited evidence of how cultural, linguistic, spiritual or socioeconomic diversity was incorporated into the design or delivery of mental health services. Students, however, consistently described these contextual factors as central to their willingness to engage with support and to their perceptions of its relevance and effectiveness. These findings suggest that achieving equitable mental health support requires more than universally available services; it also requires approaches that are culturally responsive, contextually grounded and capable of recognizing the diverse realities that shape students’ mental health experiences.

### 3.5. Informal Support Networks as Primary Sources of Care

While institutional documents focused predominantly on formal mental health services, participants consistently described informal support networks as their primary source of emotional support and coping. Peers, family members, trusted friends and, for many students, spiritual beliefs and faith communities provided accessible, trusted and ongoing sources of support that were often perceived as more responsive than formal institutional services.

Many participants explained that they turned to these informal networks because they felt understood, accepted and able to speak openly without fear of judgment. In several cases, informal support was described not simply as complementary to formal services, but as the principal mechanism through which students managed periods of psychological distress.

One participant reflected on how peer support became the most meaningful source of care after formal services had failed to meet their needs:

“… and I think that’s why we never went back, because I don’t think we got help there. I think what helped us was that we spoke about it amongst ourselves. We sort of counselled each other, yeah, it wasn’t a nice experience.”

UG3-5

In contrast, the institutional documents gave relatively little attention to the contribution of informal support networks to student wellbeing. Although peer support was occasionally referenced, it was generally presented as an adjunct to formal counselling services rather than as a central component of the university’s mental health support system. Similarly, there was limited recognition of the important roles that families, trusted social relationships and spirituality played in shaping students’ experiences of coping and resilience.

The integration of documentary and interview findings, therefore, revealed a notable divergence in how mental health support was conceptualized. Institutional frameworks primarily viewed support through the provision of formal professional services, whereas students described wellbeing as being sustained through a broader network of interpersonal relationships and community connections. These findings suggest that effective student mental health support extends beyond counselling services alone and should recognize, strengthen and meaningfully integrate informal support systems as valuable components of a comprehensive and student-centred mental health framework.

## 4. Discussion

This study explored pharmacy students’ lived experiences of mental health support within a South African university by integrating institutional document analysis with phenomenological interviews. Bringing together these complementary sources of evidence enabled the study to examine not only how mental health support was formally represented by the university, but also how it was experienced, interpreted and navigated by students in their everyday lives. The findings revealed a consistent pattern of institutional alignment in intent but dissonance in experience. While institutional documents portrayed mental health support as accessible, coordinated and inclusive, students described these same services as reactive, fragmented and insufficiently embedded within their academic and social environments. This distinction between institutional representation and lived experience represents the central contribution of this study.

The concept of institutional dissonance, described by Chandler [27] as the misalignment between organisational intentions and users’ experiences of service delivery, provides a useful framework for interpreting these findings. The document analysis demonstrated that the University had established multiple pathways for students to access mental health support, including counselling services, wellness initiatives, and crisis response procedures. However, the interview data revealed that the presence of these structures did not necessarily translate into meaningful access, engagement or trust. Rather than functioning as a coherent support system, services were experienced as peripheral to students’ everyday university lives. The integration of documentary and experiential data, therefore, illustrates the value of methodological triangulation, revealing aspects of institutional functioning that would not have been apparent through either data source alone.

A key manifestation of this institutional dissonance was the predominance of reactive responses to student distress. Participants consistently described institutional responses as triggered only when students reached a crisis point, with support frequently taking the form of academic accommodation rather than sustained psychological care. Extensions to assignment deadlines or adjustments to academic requirements were appreciated but were not viewed as addressing the underlying causes of psychological distress. Similar observations have been reported by Deasy et al. [28] and Vilhunen et al. [29], who argue that university responses often prioritize academic recovery over holistic well-being. Collectively, these findings suggest that mental health continues to be framed primarily in relation to academic performance rather than as an integral component of student development, professional identity formation and human flourishing [28,29].

The findings further demonstrated that the visibility of mental health support is as important as its availability. Institutional documents outlined referral pathways and available services, yet students frequently perceived these services as disconnected from their routine academic experiences and as becoming visible only during periods of crisis. This distinction highlights an important limitation of service-based approaches to student wellbeing. Simply providing counselling services does not ensure that students perceive these services as accessible, relevant or part of their everyday university experience. The United Kingdom Task Group on Student Mental Health Services [30] similarly argues that isolated interventions are insufficient to address the complex academic, social and environmental determinants of student mental health. Instead, universities should adopt whole-of-institution approaches that embed mental health promotion within teaching, learning, student engagement and campus culture.

The barriers identified by participants further reinforce this disconnect between institutional provision and lived experience. While university documents clearly described procedures for accessing support, they paid comparatively little attention to the practical and psychological barriers that shape students’ willingness to seek help. Long waiting times, concerns regarding confidentiality and the stigma associated with mental health emerged as significant deterrents, despite the formal availability of services. Consistent with previous qualitative research [4,12], these findings demonstrate that accessibility cannot be understood solely in structural terms. Students’ decisions to seek support are shaped by perceptions of safety, trust, confidentiality, and anticipated responses, highlighting the need for institutions to address both experiential and organisational barriers to care.

One of the most important findings of this study concerns the influence of cultural and contextual responsiveness on students’ engagement with mental health services. Although institutional documents referred broadly to inclusivity and student wellbeing, they offered limited evidence of approaches that explicitly acknowledged students’ diverse linguistic, cultural, spiritual and socioeconomic realities. Participants, however, consistently described these contextual factors as central to their experiences of mental health and help-seeking. For many students, feeling understood was closely associated with shared language, cultural familiarity and recognition of the broader social realities shaping their wellbeing. These findings suggest that culturally responsive mental health care extends beyond providing universally available services. Rather, it requires approaches that recognize the diverse ways in which students experience, interpret and communicate psychological distress. This interpretation aligns with Priestley et al. [31], who advocate for proactive, culturally responsive mental health cultures that strengthen relationships, promote early intervention and acknowledge the structural determinants of student wellbeing [31].

The prominence of informal support networks represents another important contribution of this study. Participants consistently described peers, family members and spiritual communities as trusted sources of emotional support that often compensated for perceived limitations within formal institutional services. These informal relationships were not merely supplementary; for many students, they constituted the primary means through which psychological distress was managed. In contrast, institutional documents focused predominantly on professionally delivered services and paid comparatively little attention to the role of peer relationships, family support, or spirituality in students’ wellbeing. This divergence suggests that universities may benefit from adopting broader ecological approaches to student mental health that recognize formal and informal support systems as complementary rather than competing resources. Strengthening peer mentoring, student communities and culturally meaningful support initiatives may enhance both the accessibility and acceptability of institutional mental health strategies.

Taken together, the findings suggest that the effectiveness of university mental health support cannot be judged solely by the existence of services, policies or referral pathways. Equally important is the extent to which these structures are visible, trusted, culturally responsive and meaningfully integrated into students’ everyday academic lives. By comparing institutional representations with students’ lived experiences, this study demonstrates that gaps frequently exist between organisational intentions and students’ experiences of care. Addressing this institutional dissonance requires a shift from predominantly reactive, service-oriented models towards integrated, relational and preventative approaches that position mental health as a shared institutional responsibility rather than solely an individual concern. Such approaches have the potential not only to improve access to care but also to foster learning environments that promote resilience, belonging and academic success.

This study has several limitations. It was conducted within a single university and focused exclusively on pharmacy students, which may limit the transferability of the findings to other institutional and disciplinary contexts. Nevertheless, a rich contextual description has been provided to enable readers to judge the applicability of the findings to similar settings. As with most qualitative research, the findings reflect participants’ perceptions and experiences and may have been influenced by recall bias or social desirability bias. However, integrating document analysis with interview data strengthened the credibility of the findings by enabling direct comparison between institutional representations of support and students’ lived experiences. Future research should extend this work across multiple universities and professional programs to explore how institutional context influences students’ experiences of mental health support. Longitudinal studies examining how students engage with support services over time, together with evaluations of integrated and culturally responsive mental health frameworks, would provide valuable evidence to inform policy and practice within higher education.

## 5. Conclusions

This study explored pharmacy students’ lived experiences of mental health support at a South African university by integrating institutional document analysis with phenomenological interviews. While formal mental health services were available, students consistently experienced these services as reactive, fragmented and insufficiently embedded within everyday university life. The comparison of institutional documents with students’ narratives revealed a clear pattern of institutional alignment in intent but dissonance in experience. Although the university presented mental health support as accessible and inclusive, students described significant barriers related to visibility, accessibility, cultural responsiveness, and continuity of care.

The findings demonstrate that the effectiveness of institutional mental health support cannot be judged solely by the existence of services, policies or referral pathways. Rather, meaningful support depends on how students experience, interpret and engage with these structures within the context of their academic, cultural and social lives. The study also highlights the central role of informal support networks, including peers, family and spirituality, in promoting student wellbeing, suggesting that these resources should be recognized as integral components of comprehensive mental health strategies rather than as peripheral or alternative sources of care.

By integrating institutional and experiential perspectives, this study contributes to a more nuanced understanding of student mental health support in higher education and demonstrates the value of combining document analysis with qualitative inquiry for evaluating institutional practice. The findings suggest that universities should move beyond predominantly reactive, service-based approaches towards integrated, proactive and culturally responsive models of care that embed mental health within the everyday student experience. Such approaches have the potential not only to improve access to support but also to strengthen student belonging, resilience, academic success and professional development.

Although conducted within a single South African university, the findings provide important insights for pharmacy education and for higher education institutions seeking to develop more effective and equitable mental health support systems. Future research should examine whether similar patterns of institutional dissonance exist across other universities and professional programs and evaluate interventions designed to strengthen the integration of formal and informal support within student mental health frameworks.

Ultimately, this study suggests that improving student mental health is not simply a matter of expanding services, but of ensuring that those services are visible, trusted, culturally responsive and meaningfully connected to students’ lived realities. Bridging the gap between institutional intention and students’ lived experience may be one of the most important steps towards creating universities in which mental health support is experienced not as a service on the margins, but as an integral part of student learning, wellbeing and human flourishing.

## Figures and Tables

**Table 1:** Participant demographics and unique codes. To protect the anonymity and confidentiality of participants, race was not included in the demographic table; however, the sample included one Coloured student, one Indian student, and eighteen African students. Furthermore, six of the twenty were international students from other African countries, contributing to a broader diversity of perspectives and cultural insights.

Level of Study	Participant Title	Gender	Code *
**FIRST YEAR**	Participant 1	Female	UG1-1
	Participant 2	Female	UG1-2
	Participant 3	Male	UG1-3
**SECOND YEAR**	Participant 4	Female	UG2-1
	Participant 5	Male	UG2-2
	Participant 6	Female	UG2-3
**THIRD YEAR**	Participant 7	Female	UG3-1
	Participant 8	Female	UG3-2
	Participant 9	Female	UG3-3
	Participant 10	Female	UG3-4
	Participant 11	Female	UG3-5
	Participant 12	Female	UG3-6
**FOURTH YEAR**	Participant 13	Female	UG4-1
	Participant 14	Male	UG4-2
	Participant 15	Female	UG4
**POSTGRAD**	Participant 16	Male	PG1
	Participant 17	Male	PG2
	Participant 18	Male	PG3
	Participant 19	Female	PG4
	Participant 20	Male	PG5

* Code Key: UG1-1, UG: Undergraduate, 1: First year, 1: Participant; UG2-1, UG: Undergraduate, 2: Second year, 1: Participant; UG3-1, UG: Undergraduate, 3: Third year, 1: Participant; UG4-1, UG: Undergraduate, 4: Fourth year, 1: Participant; PG1, PG: Postgraduate, 1: Participant.

## Data Availability

The full thesis with results is available on the university repository (https://researchrepository.ru.ac.za/items/e8c45145-924b-4644-aa20-004dc425f206 accessed on 26 March 2026), and any further information can be obtained from the principal researcher.

## Supplementary Materials

The following supporting information can be downloaded at: https://www.mdpi.com/article/10.3390/pharmacy14060126/s1. File S1: Interview Schedule.
