# Supplementary material for: “It’s There, but Not for Us”: Pharmacy Students’ Lived Experiences of Mental Health Support at a South African University
Source: Pharmacy (Basel). 2026 Aug 26;14(6):126. doi: 10.3390/pharmacy14060126 (PMC13610393; doi:10.3390/pharmacy14060126)
Supplement: Supplementary file 1 [file pharmacy-14-00126-s001.zip › pharmacy-4413499-supplementary.pdf]

INTERVIEW SCHEDULE:

Sample of Questions and Prompts for the research as carried out by Mr Nqobani Dabengwa (g19d1235@campus.ru.ac.za).

1. **What is your year of study, and how long have you been at Rhodes University?**

- To gauge their familiarity with Rhodes University and if that has played any role in their understanding of the mental support structure at Rhodes University.

2. **What does mental health mean to you?**
   Prompt: What is meant by the term "mental health"?
   Use probes for information on the following aspects:
   - Meaning of mental health
   - Understanding lived experiences of mental health or mental health illnesses should they disclose

3. **Could you please tell me about the mental health support structures offered by Rhodes University that you know?**

4. **Could you please share your personal experiences with the mental health support structure offered by Rhodes University if you have used it? If not, please share why.**
   Prompt: Can you please tell me how you identified the services and obtained information on them?
   Use probes for information on the following aspects:
   - What do you think led you to seek the services?
   - Which services did you use and why? If you have not, why
   - How was that experience?

   Prompt: Kindly elaborate on how you felt while navigating the whole experience.
   Use probes for information on the following aspects:
   - Cultural fitness
   - Contextual relevance
   - How did the experience leave you feeling?
